# Sex-linked transcriptional divergence in the hermaphrodite fungus *Neurospora tetrasperma*

**DOI:** 10.1098/rspb.2013.0862

**Published:** 2013-08-07

**Authors:** Nicklas Samils, Anastasia Gioti, Magnus Karlsson, Yu Sun, Takao Kasuga, Eric Bastiaans, Zheng Wang, Ning Li, Jeffrey P. Townsend, Hanna Johannesson

**Affiliations:** 1 Department of Forest Mycology and Plant Pathology, Swedish University of Agricultural Sciences, PO Box 7026, 75007 Uppsala, Sweden; 2 Department of Evolutionary Biology, Uppsala University, Norbyvägen 18 D, 75236 Uppsala, Sweden; 3 Crops Pathology and Genetics Research Unit, United States Department of Agriculture–Agricultural Research Service, Davis, CA 95616, USA; 4 Laboratory of Genetics, Wageningen University, Droevendaalsesteeg 1, 6708 PB, Wageningen, The Netherlands; 5 Department of Biostatistics, Yale University, 135 College Street, New Haven, CT 06520-8034, USA

**Keywords:** sexual dimorphism, *Neurospora tetrasperma*, gene expression

## Abstract

In the filamentous ascomycete *Neurospora tetrasperma*, a large (approx. 7 Mbp) region of suppressed recombination surrounds the mating-type (*mat*) locus. While the remainder of the genome is largely homoallelic, this region of recombinational suppression, extending over 1500 genes, is associated with sequence divergence. Here, we used microarrays to examine how the molecular phenotype of gene expression level is linked to this divergent region, and thus to the mating type. Culturing *N. tetrasperma* on agar media that induce sexual/female or vegetative/male tissue, we found 196 genes significantly differentially expressed between *mat A* and *mat a* mating types. Our data show that the genes exhibiting *mat*-linked expression are enriched in the region genetically linked to mating type, and sequence and expression divergence are positively correlated. Our results indicate that the phenotype of *mat A* strains is optimized for traits promoting sexual/female development and the phenotype of *mat a* strains for vegetative/male development. This discovery of differentially expressed genes associated with mating type provides a link between genotypic and phenotypic divergence in this taxon and illustrates a fungal analogue to sexual dimorphism found among animals and plants.

## 1. Introduction

Sexual dimorphism is the systematic difference in form between individuals of different sex in a dioecious species. Among plants and animals, sexual dimorphism is a widespread phenomenon and apparent in both gamete production and secondary sexual characteristics such as tusks, colour and body size [1,2]. Sexual dimorphism is expected to be the result of natural selection, favouring traits that increase the survival and general reproductive success of individuals of the respective sex, and/or sexual selection, favouring traits involved specifically in mating and fertilization [1,2]. The evolutionary forces that generate sexual conflicts are expected to act as an engine behind the evolution of sexual dimorphism [3]. Females and males of most dioecious animals and plants are genetically nearly identical. Thus, the majority of sexually dimorphic traits result from the differential expression of genes that are present in the genomes of both sexes [4–7].

So far, sexual dimorphism has not been recognized in the fungal kingdom. In filamentous fungi, sexual identity is governed by one or several mating-type loci [8–11], and although male and female roles and/or reproductive structures are formed during mating, phenotypic traits are with a few exceptions [12] generally exchangeable and reciprocal between mating types [11]. In outcrossing (i.e. self-sterile) filamentous ascomycetes, represented here by the model species *Neurospora crassa*, genes at the biallelic mating-type (*mat*) locus encode transcription factors that direct sex-specific gene regulatory pathways determining cell fate and identity, as well as gate-keeping sexual reproduction [10,13,14]. While *N. crassa* exhibits normal recombination along the *mat* chromosome (linkage group 1 (LGI), harbouring the *mat* locus), the congeneric species *Neurospora tetrasperma* exhibits a large (approx. 7 Mbp) region of suppressed recombination (SR) surrounding and including the *mat* locus [15–18]. The SR along the *mat* chromosomes in *N. tetrasperma* is associated with substantial DNA sequence divergence between alleles within wild-type heterokaryotic strains [19–21], in contrast to the rest of the genome, which is largely homoallelic [18–22]. Data on sequence divergence and genome architecture of multiple *N. tetrasperma* strains suggest that the suppression of recombination has manifested by expansions of the region over evolutionary time [19–21,23]. Assuming a locally conserved gene order between *N. crassa* and *N. tetrasperma* [16,17,23,24], the SR of the *N. tetrasperma mat* chromosomes encompasses over 1500 genes that are genetically linked to mating type.

Here, we demonstrate that sequence divergence of the *mat* chromosomes is accompanied by expression divergence in *N. tetrasperma.* We assayed genome-wide gene expression from multiple strains to identify genes with a mating-type biased gene expression. By correlating sequence and expression divergence, we were able to infer that genes exhibiting *mat*-linked expression are enriched in the region genetically linked to mating type, and our data show that this bias in expression correlates with sequence divergence accumulating over evolutionary time in this part of the genome. Our results indicate that the phenotype of the different mating types of *N. tetrasperma* differ in their optimal fitness values; a finding analogous to sexual dimorphism commonly found in animals and plants.

## 2. Material and methods

### (a) Fungal strains

Six strains in the genus *Neurospora* were used in this study: four strains of *N. tetrasperma* and two strains of *N. crassa*. The four strains of *N. tetrasperma* are haploid, single mating-type strains, isolated previously from two natural heterokaryotic strains (strains IDs 85 and P4492). These heterokaryotic strains of *N. tetrasperma* are closely related: they belong to the same phylogenetic lineage of *N. tetrasperma* recognized by Menkis *et al.* [22], but are genetically distinct. The two strains of *N. crassa* included in the study (IDs FGSC 2489 and FGSC 4200) are haploid and largely isogenic strains of different mating types [25]. All strains were obtained from the Fungal Genetics Stock Center (University of Missouri, MO, USA). Throughout this report, strains will be referred to by their ID and mating-type designation, i.e. 85A, 85a, P4492A, P4492a, 2489A and 4200a.

### (b) Growth conditions

For each strain, cultures were grown on two controlled nutrient agar regimes: synthetic crossing medium, which induces formation of protoperithecia, the female reproductive structures [26], and Vogel's medium (VM) [27], which, by contrast, promotes vegetative growth. The vegetative growth regime leads to the formation of vegetative mycelia but also conidia, which act as both asexual propagules and the male fertilizing unit. These two nutrient agar regimes are hereafter referred to as crossing medium and vegetative growth medium, respectively. The agar surface was covered with sterilized cellophane before inoculation, in order to facilitate tissue harvest. All strains were simultaneously cultured on both nutrient sources in darkness at 25°C for 5 days. Immediately after harvest, tissue was frozen at −80°C.

### (c) Microarray experimental design

**Figure 1.**
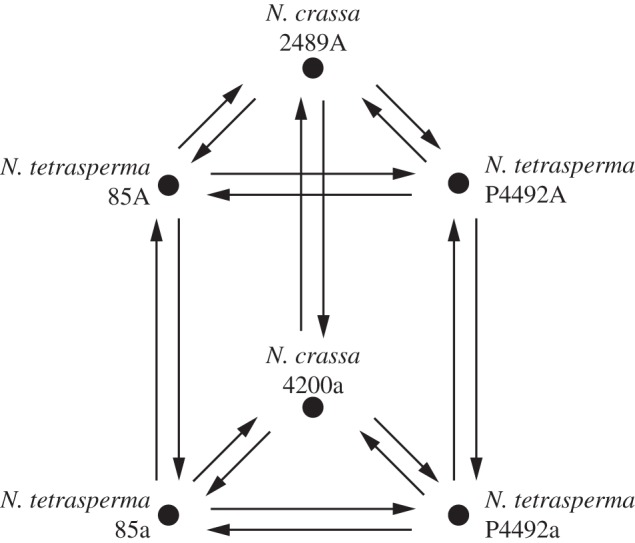
Experimental design for the microarray study. Nodes in the multigraph represent samples of mRNA isolated from the six homokaryotic *Neurospora* strains. The arrows indicate dye-swap hybridizations (Cy3 and Cy5). The same set-up was used for independent microarray hybridizations with RNAs from strains grown on crossing medium and on vegetative growth medium.

The hybridization scheme for the microarrays followed the guidelines of Townsend & Taylor [28] and is depicted in figure 1. We separately studied the expression profiles of *Neurospora* strains grown on crossing medium and vegetative growth medium, so as to identify patterns of expression divergence that are specific for tissue types and/or composition of the medium [29]. In order to categorize the collection of differentially expressed genes into transcriptional differences that are mating-type specific and strain specific, we included two pairs of *mat A* and *mat a* strains of *N. tetrasperma* in the experimental design (cf. [30]). In addition, one pair of *mat A* and *mat a* strains of *N. crassa* was included with the aim to identify a set of genes that are also regulated by mating type in a species of *Neurospora* with no suppression of recombination around the *mat* locus, i.e. expression differences that are attributable only to the *mat* locus. Every sample was included in six independent measurements (represented by arrows in figure 1), which resulted in 18 independent hybridizations per nutrient regime.

### (d) RNA extraction and microarray hybridization

All RNA extractions were performed following the protocol of Clark *et al.* [31]. In brief, total RNA was extracted separately from pooled cultures of each strain grown on crossing medium or vegetative growth medium by using TRI REAGENT (Molecular Research Center Inc. Cincinnati, OH, USA). Tissues were homogenized with a Dounce glass grinder and debris filtered through Qiagen Qiashredder columns (Qiagen, Chatsworth, CA, USA). Poly(A)^+^mRNA was obtained from these extractions by using MRColigo (dT) columns (Molecular Research Center, Inc.). The cDNA synthesis was based on 2 μg poly-A mRNA, using Superscript II (Invitrogen) and 0.5 µg oligo (dT). Coupling was performed with Cy3 and Cy5 labelled probes (Amersham Biosciences, Uppsala, Sweden), and competitive hybridizations were performed in the dark at 55°C for 16 h. Hybridizations were performed on whole-genome-spotted oligonucleotide microarrays designed for *N. crassa*, featuring 70 mer oligonucleotides uniquely representative of 9826 open reading frames, robotically printed on CMT-GAPS -aminopolysilane-coated glass slides (Corning, Corning, NY, USA) at the Yale University Center for Genomics and Proteomics, as in Kasuga *et al*. [32]. Since we focused on differences within species, we consider that this array, although designed on *N. crassa*, is appropriate for expression analyses of both *N. crassa* and *N. tetrasperma*. Moreover, previous studies have shown that sequence variability between *N. crassa* and *N. tetrasperma* is very low, with the proportion of variable sites of nuclear genes being 5 per cent or less [19]. We cannot exclude the possibility that sequence differences between strains of different mating type in *N. tetrasperma* have accumulated at the probe sequence sites, possibly resulting in false positive estimations of expression divergence, and/or that we underestimate the differences between *N. tetrasperma* mating types, since some genes in divergent areas, especially short genes, might not have been detected with the *N. crassa* probes used for this study [33]. However, the strength of our approach is that we use the *N. crassa* microarray to emphasize differences in gene expression among *N. tetrasperma* strains, each of which are equally diverged to *N. crassa* (see [21] for a genomic comparison of P4492A, P4492a and *N. crassa*).

### (e) Image acquisition

The microarray slides were scanned by a Genepix 4000B scanner (Axon Instruments, Foster City, CA, USA), providing a two-channel, digital 5 µm colour image. The software Genepix Pro v. 6.0 (Axon Instruments, CA, USA) was applied with the array list file located on http://www.yale.edu/townsend/Links/ffdatabase/downloads.html to locate spots and quantify the hybridization signal strengths in each channel. Genes were deemed as expressed when the median spot foreground exceeded the median background plus two standard deviations of the background intensity. All microarray hybridization images were manually inspected for hybridization quality and adjusted to acquire optimal images before data collection. Abnormal spots were excluded from the analysis.

### (f) Expression data analyses

Expression ratios were retained, and the acquired datasets were normalized as described in Townsend *et al.* [34] and at http://bioinfo.townsend.yale.edu/online.jsp. To estimate relative expression levels and 95% credible intervals for each gene in each sample, Bayesian analyses of gene expression levels (BAGEL) [35] was performed using the version UBAGEL 3.6, separately on normalized data from the two nutrient medium conditions. Accession numbers for all genes that exhibited expression signal and could be analysed were retrieved from the MIPS (Munich Information Center for Protein Sequences) database (http://mips.helmholtz-muenchen.de/genre/proj/ncrassa/). For subsequent analyses, we focused primarily on the genes that show a mating-type biased expression in *N. tetrasperma*, i.e. those that are expressed in strains of both mating types but consistently show a significantly higher expression level in one of the mating types. The genes with absence of expression in one of the samples during competitive hybridization were automatically removed from the BAGEL analyses, and thus not included in the study.

### (g) Quantitative RT-PCR experiments

For a subset of genes, the relative expression from the microarray analysis was verified with quantitative PCR on reverse-transcribed RNA (qRT-PCR). Reverse transcription of the same mRNA samples used for the microarray experiment was primed using an oligo-dT primer and the iScript cDNA synthesis kit (Bio-Rad, Hercules, CA, USA). Transcript levels were quantified by qPCR as previously described [36] using primers listed in the electronic supplementary material, table S1. Expression levels were calculated according to the 2^−*ΔΔ*Ct^ method [37], using gene expression level of actin (*act*; not differentially expressed between any of the strains in this study) for normalization, as in Karlsson *et al*. [38]. Pearson correlation estimates for the comparison between microarray and qRT-PCR data were calculated in R.

### (h) Genomic distribution of mating-type biased genes

We mapped the distribution of the *N. tetrasperma* mating-type biased genes into chromosomes and chromosomal regions. For this analysis, we assumed a locally conserved gene order between *N. crassa* and *N. tetrasperma* [16,17,23,24] (http://www.broadinstitute.org/annotation/genome/neurospora/MultiHome.html). We used the divergence data on strain P4492 from previous studies [20,21] to delimit the region of sequence divergence on the *mat* chromosome in this lineage (including in total 1618 genes). Based on the threshold of 1 per cent sequence divergence (cf. [21]), we partitioned the *N. tetrasperma* mating-type biased genes into the SR and the pseudoautosomal (PA) flanking regions of the *mat* chromosome (linkage group (LG)I_SR and LGI_PA, respectively), and on the six autosomes (corresponding to LGs LGII-LGVII in *N. crassa*). Genes whose genomic location was mitochondrial or unknown were grouped into a separate category, ‘LG_Other’. To evaluate enrichment of the mating-type biased genes within chromosomes and chromosomal regions, we performed a Fisher one-tailed analysis using an in-house R (http://www.r-project.org/) script and the *Q*-value package [39] for multiple testing using the bootstrap method described in [40]. Furthermore, to investigate whether *N. tetrasperma* mating-type biased genes were clustered within any of the chromosomes, we applied model-averaged clustering by maximum-likelihood (MACML) [41] to the intrachromosomal sequences of differentially expressed and not differentially expressed genes. MACML applies a tripartite divide-and-conquer maximum-likelihood algorithm, providing the greatest available power and precision for detecting heterogeneous clustering within discrete linear sequences [41]. To avoid over-parametrization, we penalized cluster likelihoods using the Bayesian information criterion.

### (i) Correlation between sequence divergence and expression divergence for strain P4492

Medium coverage genomic sequence of the two haploid genomes P4492A and P4492a was recently released [21,42]. We generated consensus genome sequences of the two isolates by reference assembly using *N. crassa* as reference, as described in Sun *et al.* [21]. For each window of 200 kb along the *mat* chromosome, we estimated the sequence divergence between the genomes as the fraction of nucleotides that differed between them, and expression divergence as the fraction of genes (out of the total number of genes in that window for which we obtained a microarray signal of expression) that were significantly mating-type biased during at least one of the two growth regimes.

## 3. Results and discussion

In this study, we used a combination of genomic and microarray tools to investigate *mat*-linked gene expression divergence in the fungus *N. tetrasperma*. Following data treatment and mapping of microarray probes to gene identifiers, a total of 6159 and 4048 genes were identified as expressed when tissue was grown on crossing and vegetative growth medium, respectively. The higher number of genes expressed in sexual/female tissue can be attributed to the increased complexity of transcription across *N. crassa* sexual development [29] in comparison with vegetative/male development [43]. In our samples, in particular, the sexual tissues constitute both hyphae and protoperithecia of different stages of maturity. The gene expression data and functional annotations have been deposited to the Filamentous Fungal Gene Expression Database [44] (Experiment ID 43; http://bioinfo.townsend.yale.edu) and to the NCBI Gene Expression Omnibus data repository (http://www.ncbi.nlm.nih.gov/geo/index.html).

### (a) Mating-type biased gene expression in *Neurospora*

**Figure 2.**
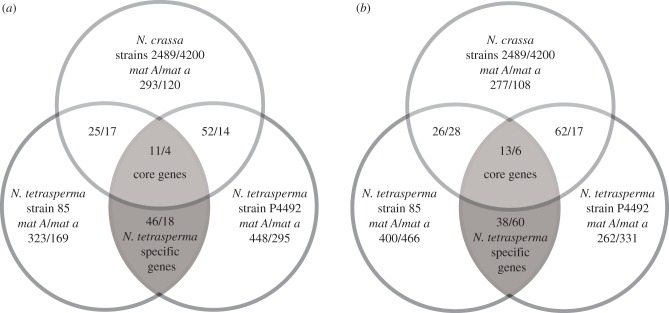
Venn diagrams showing the number of differentially expressed genes (*p* < 0.05), between *Neurospora* strains of different mating type (upregulated in the *mat A* strain/upregulated in the *mat a* strain) in *N. crassa* and in *N. tetrasperma*. Each diagram shows the number of genes that are specifically differentially expressed in each medium, (*a*) crossing medium and (*b*) medium inducing vegetative growth. Numbers in overlapping regions represent genes differentially expressed in more than one pair of strains of different mating type: grey areas represent the *N. tetrasperma* mating-type biased genes, and this is subdivided into the core *Neurospora* mating-type biased genes (upper grey areas) and the *N. tetrasperma* specific mating-type biased genes (lower grey areas).

By analysing multiple closely related strains of *Neurospora*, we were able to extract a set of genes that are consistently differentially expressed between strains of different mating type. The numbers of genes found by Bayesian analyses to be significantly differentially expressed (*p* < 0.05) between *mat A* and *mat a* strains of *N. tetrasperma* and *N. crassa*, grown under the two nutrient regimes, are depicted in figure 2. Summing up over both tissue types, 196 genes exhibited a mating-type biased expression in *N. tetrasperma*, i.e. they were consistently differentially expressed between *N. tetrasperma* strains of different mating type (see figure 2 and the electronic supplementary material,  table S2). Included in this number are the ‘core’ *Neurospora* mating-type biased genes (the upper shaded areas of the Venn diagrams of figure 2) and the *N. tetrasperma* ‘specific’ mating-type biased genes (the lower shaded areas). These gene categories can be further subdivided into *mat A-* and *mat a*-biased genes, depending upon which mating type shows the higher expression (see figure 2 and the electronic supplementary material, table S2). We consider our approach to identify mating-type biased genes to be conservative, since the exclusion of the genes with absence of expression in strains of one mating type from the analyses is likely to lead to an underestimation of the mating-type biased genes. These genes may be of potential importance for phenotypic differentiation of mating types and should therefore be prioritized for further studies on this topic. The robustness of our findings using microarrays was supported by qRT-PCR on a selection of genes (see the electronic supplementary material, table S4). Magnitudes of gene expression (fold changes) between mating types, as estimated by qRT-PCR, were higher than when estimated through the microarray hybridizations (see the electronic supplementary material, table S4), as is typically expected [45]. There was a significant (*p* = 0.00067) positive correlation (*R* = 0.66) between expression ratios from the two approaches as revealed by a Pearson correlation test.

The mating-type linked transcriptional divergence identified herein indicates that the SR along the *mat* chromosome in *N. tetrasperma* is accompanied by transcriptional divergence in this filamentous ascomycete. The finding that the two mating types of *N. tetrasperma* most probably contain the same set of genes [23], implies that in fungi, as well as in animals and plants, differences in gene regulation may be an important contributor to phenotypic dimorphism [4,5,46–48].

### (b) Candidate genes involved in mating-type dimorphism

The differences in gene expression demonstrated in this study identified candidate genes associated with phenotypic differences between *mat A* and *mat a* strains in *N. tetrasperma* (electronic supplementary material, table S2). For example, two key genes for sexual reproduction in *Neurospora,* the pheromone receptor *pre-1* and pheromone precursor *ccg-4* (in both of which expression is directly influenced by the *mat A-1* (see [49], and references therein)) were all found significantly upregulated in all three *mat A* strains grown on crossing medium, i.e. they were a part of the *Neurospora* core genes (electronic supplementary material, table S2). Although none of the known sexual or pre-sexual genes were found to be upregulated in *mat a* strains, sequence analysis of two hypothetical proteins (MIPS: NCU03180 and NCU21864) induced in all *mat a* strains grown on crossing medium suggested roles for these uncharacterized genes in sexual reproduction: NCU03180 is predicted to be secreted through a non-classical secretion pathway, while NCU21864 is predicted to be integrated into the plasma membrane, exposing a considerable part of the protein to the outside environment (see the electronic supplementary material, table S2). The fact that these proteins have a putative extracellular localization suggests that the two proteins are exposed to the cell surface and might play a role in cell–cell communication or mating recognition. It is tempting to suggest that these genes may encode for yet unknown pheromones and/or pheromone receptors in *Neurospora*; functional characterization of deletion mutants for the two genes in the model species *N. crassa* could experimentally confirm this hypothesis.

### (c) The accumulation of the mating-type biased genes on the mating-type chromosomes

**Figure 3.**
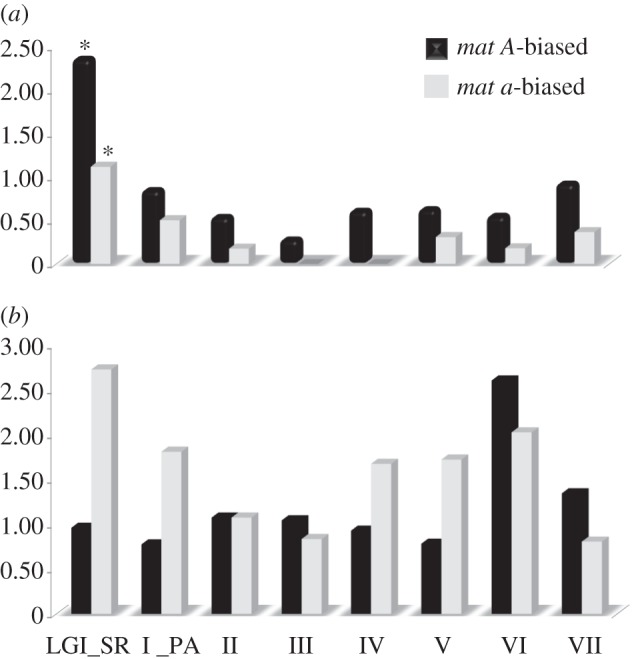
Chromosomal partitions of the *N. tetrasperma* mating-type biased genes (i.e. genes consistently differentially expressed between *N. tetrasperma* strains of different mating type). Genes upregulated in *mat A* strains are represented by black bars and *mat a* by grey bars, data from strains grown on crossing medium are shown in (*a*), and on vegetative growth medium in (*b*). Bar heights represent percentage of the total number of genes that are expressed (i.e. detected by BAGEL analysis) that are mating-type biased in each chromosomal region (*x*-axis, linkage groups I–VII). For the *mat* chromosome, genes are partitioned into genes located in the region of suppressed recombination (I_SR) and into genes located in the freely recombining flanks of the chromosome (I_PA). Asterisks represent significant enrichment, i.e. *Q*-value of Fisher ≤ 0.05.

We found that when *N. tetrasperma* is grown on crossing medium, the mating-type biased genes are enriched on the *mat* chromosome (see figure 3*a* and the electronic supplementary material, table S5). Under this growth regime, a relatively high proportion of the *N. tetrasperma* mating-type biased genes locates in the region of SR on the *mat* chromosome (LGI_SR, figure 3*a*), i.e. the region that is genetically linked to the mating-type locus. Fisher analysis for enrichment of genomic locations showed that the enrichment is statistically significant in genes biased for both mating types (*Q* < 0.05), although the signal is especially strong in genes with *mat A*-biased expression (figure 3*a*). Furthermore, the signal of significant enrichment remains when only *N. tetrasperma* specific mating-type regulated genes were analysed (data not shown). This finding is similar to the enrichment of male-biased genes on sex chromosomes found in certain mammals [50–52], although a pattern of the accumulation of sex-biased genes on sex chromosomes is not generally confirmed [6].

By contrast, for mating-type biased genes identified during vegetative growth, no significant chromosomal enrichment was found: genes that were *mat a*-biased during vegetative growth were found in high proportions on LGI_SR and LGVI (figure 3*b*), but this enrichment was not significant. The *N. tetrasperma mat A*-biased genes during vegetative growth showed a high relative occurrence on LGVI, but again, this pattern was not significant (figure 3*b*). The lack of significance for the enrichment of the genes expressed during vegetative growth is most probably owing to the small number of differentially expressed genes.

### (d) A positive correlation between expression and sequence divergence

**Figure 4.**
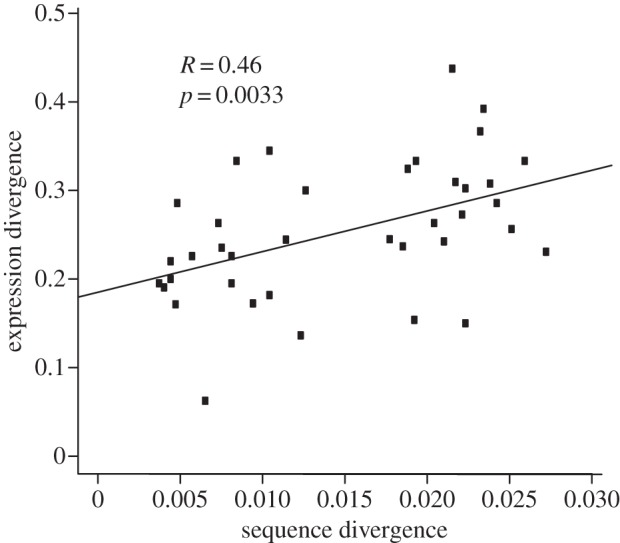
The correlation of sequence and expression divergence of genes located on the *mat* chromosome of *N. tetrasperma*.

A positive correlation of the expression and sequence divergence across the *mat* chromosome suggests that in *N. tetrasperma*, the mating-type bias in gene expression has accumulated as a consequence of sequence divergence. Specifically, we plotted within 200 kb sliding windows on the *mat* chromosome, the expression divergence (the fraction of genes that are differentially expressed between *A* and *a* strains relative to all expressed genes) versus the sequence divergence (the fraction of nucleotides that differed between *A* and *a* strains), and calculated the Pearson's correlation (figure 4). A significant positive correlation between sequence and expression divergence was detected (*R* = 0.46, *p* = 0.0033). Our analyses of spatial patterns in the accumulation of expression changes revealed that within each chromosome and chromosomal region, genes regulated by mating type in *N. tetrasperma* were generally evenly distributed. Visually, no large-scale clustering appears in relation to the sequence divergence plot of P4492 on chromosome 1 (see the electronic supplementary material, figure S1). Statistical testing for clustering *via* maximum likelihood on all chromosomes [41] yielded two statistically significant clusters on chromosome 4 (LGIV) for genes induced in *mat a* strains grown on vegetative growth medium, and one statistically significant cluster on LGV for genes induced in *mat A* strains grown on crossing medium (see the electronic supplementary material, table S6). For genes showing *mat A*-biased expression when strains were grown on vegetative growth medium and *mat a* strains grown on crossing medium, no clusters of differentially expressed genes were detected. The absence of clusters of genes on the *mat* chromosome indicates that changes in gene expression are the result of small and independent changes, potentially associated with the accumulation of *cis*-regulatory mutations linked to mating type. In this context, it is worth to point out that we, in a recent study [21], found inconclusive indications of introgression of DNA on the *mat a* chromosome of *N. tetrasperma* strain P4492, and thus we cannot be certain that the accumulation of mutations along the chromosomes has taken place within the genome of P4492, or within another, closely related lineage or species.

### (e) The evolutionary causes of mating-type biased gene expression

The findings of a positive correlation between sequence and expression divergence and the absence of physical clustering of *mat*-regulated genes would be consistent with mating-type dimorphism in *N. tetrasperma* evolving under neutrality, i.e. following a molecular clock (cf. [53,54]). However, the findings are also compatible with alternative scenarios, whereby selected traits with *mat*-linked divergence influence the fitness of strains of different mating type. In animals, traits such as growth rate, thermoregulation, metabolic rate, biorhythms and water balance differ between sexes in their optimal value [55,56], and most of the phenotypic differences observed between sexes are thought to be the result of natural and/or sexual selection on traits that influence the fitness of each sex [57–59]. This causation could be applicable in *N. tetrasperma*: an early study by Howe [60] showed that, in this species, a series of macroscopically distinguishable characteristics, related to both asexual and sexual reproduction, are associated with mating type. These traits were mycelial pigmentation, colour of conidia (asexual propagules/male fertilizing units), and size and frequency of the protoperithecia [60]. Furthermore, and in line with a model of selection for sexual dimorphic traits in *N. tetrasperma*, our data on mating-type biased gene expression indicates large-scale patterns of expression divergence associated with growth on different nutrient regimes: we observed an excess of *mat A*-biased genes when grown on crossing medium, inducing protoperithecia formation, while the majority of the genes expressed when grown on vegetative medium, inducing formation of mycelium and conidia, are *mat a*-biased (figure 2). This finding indicates that the phenotype of *mat A* strains is primarily optimized for traits promoting sexual/female development and the phenotype of *mat a* strains for vegetative/male development. This result is in agreement with divergent roles of mating types in pre-sexual development and crossing suggested from the study of genome-wide expression profiles during asexual development in *N. crassa* [43]. Furthermore, it agrees with our results from functional categorization of mating-type biased genes: the FunCat catalogue from MIPS was used to associate genes with described function [61,62] as described in Ruepp *et al.* [62] (see the electronic supplementary material, table S3), and the analysis shows that *mat A*-biased genes are enriched in functional categories involved in mating, whereas the categories for which *mat a*-biased genes were enriched were of a housekeeping nature. All the above indicates that sex-specific election is likely to be a factor driving the divergence in *N. tetrasperma*. Finally, the enhanced accumulation (i.e. the higher percentage of the total number of genes that are expressed) of *mat A*-biased genes on the *mat* chromosome when grown on crossing medium (figure 3*a*) and of *mat a*-biased genes when grown on VM (figure 3*b*), indicates that the response to sex-specific selection in strains of different mating types in *N. tetrasperma* has resulted in feminization of the *mat A* chromosome and masculinization of the *mat a* chromosome; analogous to the predictions of the influence of sex-specific selection on the early evolution of sex chromosomes in *Drosophila* [63].

One may also speculate that the evolution of mating-type dimorphism in *N. tetrasperma* could be affected by mating-type antagonism, i.e. a conflict arising from traits that are beneficial to strains of one mating-type but harmful to the other. In *Neurospora*, as in most filamentous ascomycetes, determination of sexual identity by alternative mating-types differs from animals and plants in that it takes place at the haploid stage. *Neurospora tetrasperma* grows in nature as heterokaryotic for mating type, but the association is not complete; *N. tetrasperma* produces both sexual and asexual spores containing nuclei of only one mating type [64–66] and it has a recombining population structure suggesting the existence in nature of homokaryotic, single mating-type strains [22,67]. This character results in limited opportunity for antagonism between mating-type linked genes/alleles. By contrast, mating-type antagonistic genes/alleles are likely to be found on the autosomes, and may go to fixation if the benefits for one mating-type exceed the costs incurred by the other, as expected for a sexual antagonistic gene/allele located on an autosome in a diploid organism with male or female heterogamety [6]. During heterokaryotic growth, mating-type differences in the optimal phenotype imply that some alleles can favour nuclei of one mating type at the expense of the other mating-type and/or the heterokaryon. Here, the outcome depends on the association between the nuclei in the heterokaryon. At present, precise knowledge on the association of nuclei of different mating types in nature is lacking for *N. tetrasperma*, and further investigations are needed in order to reveal internuclear dynamics of the heterokaryotic mycelium. Furthermore, the intramycelial dynamics of the nuclei in a heterokaryon, such as whether the phenotype of the heterokaryotic mycelia reflects the collective genotype of the different nuclei, is also largely unknown in *N. tetrasperma*. Taken together, our data suggest that the gene expression divergence in *N. tetrasperma* may have accumulated neutrally in association with sequence divergence, but potential selection pressures for mating-type dimorphic traits, and mating-type antagonism, deserve continued consideration.

## 4. Concluding remark

In this study, we present strong evidence for mating-type biased gene expression in the filamentous fungus *N. tetrasperma*. We show that mating-type biased genes are enriched on the *mat* chromosome, and the divergence in expression is positively correlated with sequence divergence of the *mat* chromosomes. Thus, the data presented here provide a link between genotypic and phenotypic divergence in this species. Our data indicate that expression divergence is closely associated with accumulation of *cis*-regulatory mutations and suggest that the phenotypes of the different mating types of *N. tetrasperma* differ in their optimal values; a finding analogous to sexual dimorphism commonly found in animals and plants. Our study provides a list of candidate genes potentially important for sexual dimorphism in this hermaphroditic fungus, and serves as a foundation to study the evolutionary significance of mating-type dimorphism. For example, high priority directions for future studies are within-species variability of genes with dimorphic expression and the evolutionary rate of genes expressed specifically in male and female tissues, preferably addressed by next-generation sequencing of the genomic and transcriptomic datasets. Although it is clear that sexual dimorphism does evolve, the genetic mechanisms and evolutionary forces it involves remain still poorly understood [68]. Therefore, the system presented in this study provides novel and complementary approaches for studying this phenomenon.
